# Increased habitual flavonoid intake predicts attenuation of cognitive ageing in twins

**DOI:** 10.1186/s12916-021-02057-7

**Published:** 2021-08-23

**Authors:** Amy Jennings, Claire J. Steves, Alexander Macgregor, Tim Spector, Aedín Cassidy

**Affiliations:** 1 Institute for Global Food Security, Queen’s University Belfast, Belfast, Northern Ireland; 2 Department of Twin Research & Genetic Epidemiology, King’s College London, St Thomas’ Campus, London, UK; 3 Norwich Medical School, University of East Anglia, Norwich, UK

**Keywords:** Diet, Flavonoids, Cognitive ageing, Brain volume

## Abstract

**Background:**

Although the pathophysiology of cognitive decline is multifactorial, and modifiable by lifestyle, the evidence for the role of diet on cognitive function is still accumulating, particularly the potentially preventive role of constituents of plant-based foods.

**Methods:**

We aimed to determine whether higher habitual intake of dietary flavonoids, key components of plant-based diets, were associated with improved cognition and medial temporal lobe volumes using three complementary approaches (longitudinal, cross-sectional and co-twin analyses). In 1126 female twins (*n*=224 with a 10-year follow-up of diet and cognition data) aged 18–89 years, habitual intakes of total flavonoids and seven subclasses (flavanones, anthocyanins, flavan-3-ols, flavonols, flavones, polymeric flavonoids (and proanthocyanidins separately)) were calculated using validated food frequency questionnaires. Cognition was assessed using the Cambridge Neuropsychological Test Automated Battery test. Hippocampal volumes were measured in a subset using magnetic resonance imaging (16 monozygotic-twin pairs). Statistical models were adjusted for a range of diet and lifestyle factors.

**Results:**

Higher intakes of flavanones (tertile (T)3-T1=0.45, 95%CI 0.13,0.77; *p*=0.01) and anthocyanins (T3-T1=0.45, 95%CI 0.08,0.81; *p*=0.02) were associated with improvements in age-related cognition score over 10 years. In cross-sectional analysis higher intake of flavanones (T3-T1= 0.12, 95% CI 0.02, 0.21; *p*=0.02) and proanthocyanidins (T3-T1= 0.13, 95% CI 0.02, 0.24; *p*=0.02) were associated with improved paired-associates learning. Higher intake of anthocyanins was significantly associated with improved executive function (T3-T1= −0.52, 95% CI 0.19, 0.84; *p*=0.001) and with faster simple reaction times (T3-T1= −18.1, 95% CI −35.4, −0.7; p=0.04). In co-twin analysis, those with higher anthocyanin (2.0%, *p*=0.01) and proanthocyanidin (2.0%, *p*=0.02) intakes at baseline had the largest left hippocampal volumes after 12 years.

**Conclusion:**

Small increases in habitual intake of flavonoid-rich foods (containing anthocyanins, flavanones and proanthocyanidins; equivalent to approximately two servings of oranges and blueberries per day) over long time periods have the potential to attenuate cognitive ageing.

**Supplementary Information:**

The online version contains supplementary material available at 10.1186/s12916-021-02057-7.

## Background

There is increasing evidence that dietary flavonoids, part of a diverse range of polyphenolic compounds present in plant-based foods, may be beneficial for cognitive health. In older adults, a higher habitual intake of berries was associated with slower rates of cognitive decline, with regular blueberry consumption delaying cognitive ageing by 2.5 years [1]. Higher baseline total flavonoid intakes were associated with reduced cognitive decline over 10 years [2] and improved executive function in adults with mild to moderate Alzheimer’s disease [3]. In contrast, total flavonoids were not associated with cognitive change over 6 years in a Spanish prospective cohort study [4]. Higher intakes of flavonols, anthocyanins and flavonoid polymers were associated with a lower risk of Alzheimer disease, and related dementias in the Framingham Offspring Cohort [5] and higher intakes of flavan-3-ols, flavonols and anthocyanins were positively associated with cognitive status, assessed using the Short Portable Mental Status Questionnaire [6].

Mechanisms proposed for the neuroprotective effects of dietary flavonoids and their downstream metabolites include modulating neuronal signalling pathways associated with synaptic plasticity, reducing neuroinflammation and improving cerebrovascular blood flow with particularly strong mechanistic evidence for the flavonoid sub-classes flavanols, anthocyanins and flavanones [7, 8]. Many flavonoid metabolites can cross the blood-brain barrier with recent evidence suggesting these metabolites may also be a mediator of the microbiome-gut-brain-axis [9]. In animal models, supplementing the diet with blueberries increased levels of anthocyanin and flavan-3-ol metabolites in the brain which were associated with enhanced spatial working memory via effects on the ERK-CREB-BDNF neuronal signalling pathway [10]. In healthy adults, cerebral blood flow was increased following consumption of drinks high in flavan-3-ols (494 mg) [11], anthocyanins (387 mg) [12] and flavanones (70.5 mg) [13].

Limited evidence from epidemiological studies suggests that total flavonoid intake [2, 3] and berries [1] are associated with specific cognitive domains. In short-term human intervention studies, intakes of berries [14, 15], cocoa flavan-3-ols [16] and flavanone-rich orange juice [17] have shown to exert beneficial effects on episodic memory, visual memory, executive function and psychomotor function, in addition to an attenuation of overall cognitive decline. Short-term animal dietary interventions with cocoa flavonols [18], blueberries [19] and anthocyanins [20] also report improved regional hippocampal function and hippocampus-dependent memory. On the basis of this data, we hypothesised that higher intakes of flavonols, flavan-3-ols, anthocyanins and flavanones would be associated with improved cognition.

The aim of the current study was to explore, for the first time, the associations between habitual intakes of total flavonoids and a wide range of flavonoid subclasses with biomarkers of cognition and brain volumes in a cohort of healthy, female twins using longitudinal, cross-sectional and co-twin data. Due to the variability in biological availability, mechanisms of action and bioactivity of the different flavonoid sub-classes (flavonols, flavones, flavanones, flavan-3-ols, anthocyanins and flavonoid polymers (proanthocyanidins and other polymers)), we examined each subclass separately. Our primary aim was to examine the associations between 10-year trajectories of intakes of these different flavonoid subclasses and biomarkers of cognition. This novel twin dataset allowed us to examine these associations independently of genetic variation and residual environmental confounding. In a sub-study, we investigated novel longitudinal associations between flavonoid sub-class intakes and medial temporal lobe structures.

## Methods

### Study population

Participants who were included in these analyses were female twins who were enrolled in the TwinsUK registry, which is a nationwide registry of UK adult twins who were recruited from the general population [21]. This is a female cohort as historically the study was predominantly focused on diseases with a higher prevalence in females (osteoporosis and osteoarthritis). All participants were unaware of the specific hypotheses being tested and were not selected for particular diseases or traits. All twin pairs were reared together. The participants have been shown to be representative of the general female population in terms of disease-related characteristics and dietary intake [22, 23]. The study was approved by the St. Thomas’ Hospital Research Ethics committee, and all subjects provided informed written consent.

In total, 5772 female twins completed at least one food frequency questionnaire (FFQ) between 1999 and 2017, of whom 17% (*n* = 999) were excluded for incomplete records (10 food items left blank) or for having reported an implausible energy intake (defined as the ratio of energy intake to estimated basal metabolic rate having fallen 2 SDs from the population mean). Of these participants, *n* = 1126 (*n* = 497 twin pairs, and *n* = 132 individuals without a co-twin) attended a clinical assessment for cognitive testing at the same time and are included in the cross-sectional analysis and *n* = 224 (*n* = 108 twin pairs, and *n* = 8 individuals without a co-twin) completed a FFQ and attended a clinical assessment for cognition in 1999 (aged 40–72 years) and again in 2009 (aged 50–81 years) and are included in the longitudinal analysis. In sub-set analysis, 16 monozygotic twin pairs (aged 42–69 years) who completed a FFQ in 1999 and attended for magnetic resonance imaging (MRI) 12 years later in 2011–2012 were included, as previously described [24] (Supplemental Figure 1).

### Assessment of cognition and brain volumes

Cognition was measured using CANTAB (Cambridge Neuropsychological Test Automated Battery) tests which were combined using principal component analysis to assess age-related change in global cognition; the full details of the tests and the generation of the composite cognition score have been described previously [25]. The CANTAB battery has been standardised among a predominately female cohort of older adults (55–80 years) [26]. We selected the CANTAB tests that assessed the cognitive domains considered to be sensitive to flavonoid intake in previous analysis and mechanistic studies [14, 16]. These domains were episodic memory (paired-associates learning, PAL), visual memory (delayed matching to sample, DMS), executive function (intra/extradimensional shift (IED)) and psychomotor function (simple reaction time, SRT). Prior to completing the CANTAB battery, participants underwent a motor-screening training test to introduce them to the equipment and help them relax.

In a subset of 16 MZ twin pairs, MRI scans were acquired using the same 1.5-tesla Signa HDx MR scanner (GE Medical Systems, Milwaukee, Wisconsin, USA), full details of which have been previously published [24]. In the current study, our region of interest was the medial temporal lobe incorporating the hippocampus and parahippocampus, identified using a mask from the Automated Anatomical Labelling library. Associations between brain structure and cognitive function have previously been reported in this cohort [24].

### Assessment of flavonoid intakes

Participants completed a 131-item FFQ [27]. Flavonoid values were assigned to each of the foods listed in the FFQ, and for recipes, a value for each ingredient in the dishes was assigned using data from the US Department of Agriculture (USDA) as the primary data source [28, 29] or phenol explorer (www.phenol-explorer.eu) where data were not available to ensure that all available high-quality data on flavonoid values were included. As FFQs are designed to rank participants rather than provide quantitative estimates, intakes were categorised into tertiles for all analyses.

Intakes were derived for the main subclasses of flavonoids habitually consumed as mg/d: flavanones (eriodictyol, hesperetin and naringenin), anthocyanins (cyanidin, delphinidin, malvidin, pelargonidin, petunidin and peonidin), flavan-3-ols (catechins and epicatachins), flavonols (quercetin, kaempferol, myricetin and isohamnetin), flavones (luteolin and apigenin), polymeric flavonoids (including proanthocyanidins (excluding monomers), theaflavins and thearubigins) and proanthocyanidins (dimers, trimers, 4–6 mers, 7–10 mers, polymers and monomers). Total flavonoid intakes were derived by the addition of the six component subclasses (excluding the separate proanthocyanidins class). Intakes of isoflavonoes were not calculated due to the low consumption of isoflavone-containing foods in the UK population [30]. We have previously reported the main food sources of the main subclasses of flavonoids in this cohort [31]. In analysis of the FFQ, we applied a correction to the following fruits: peaches, plums, apricots and berries, to account for consumption during the summer season by dividing portion sizes by three.

### Assessment of covariates

Information on education, occupation, smoking, supplement use and menopausal status was obtained by a standardised nurse-administered questionnaire. Education level was self-reported as the highest academic credential received and grouped into four categories. The estimated years of education for these four categories are (1) no qualifications (10 years); (2) O-Level, GCSE, NVQ2/SVQ2, or Scottish Intermediate (12 years); (3) Scottish Higher, NVQ3, city and guilds, Pitman, A Level, Scottish Advanced Higher, or Higher Vocational training (14 years); and (4) University degree, Postgraduate degree, NVQ5, or SVQ5 (17 years) [32]. Physical activity was classified as inactive, moderate and active during work, home and leisure time using a questionnaire strongly correlated in this cohort with more in-depth assessment recording how much time subjects spent in moderate and vigorous non–weight-bearing and weight-bearing activity, on average per week [33]. Intakes of energy and other nutrients were determined from the FFQ previously described using values from the UK national food composition tables [34]. Height was measured to the nearest 0.5 cm with the use of a wall-mounted stadiometer, weight (light clothing only) was measured to the nearest 0.1 kg with digital scales, and body mass index was calculated (kg/m^2^). Verbal IQ score was predicted from the number of errors made on the National Adult Reading Test (NART) using the equation (129.0−(0.919 * NART errors). Zygosity was ascertained by questionnaire and confirmed via subsequent genotyping as part of genome-wide association studies (PE Applied Biosystems, Foster City, California).

### Statistical analysis

First, in longitudinal analysis, we calculated a 10-year change in age-related change (ARC) score and examined associations with tertiles of a 10-year change in intake of flavonoid subclasses. The change was calculated by subtracting baseline intakes from follow-up values so positive values indicated higher intakes at follow-up in comparison to baseline. The CANTAB tests chosen for inclusion in the ARC score examined memory and processing speed as these cognitive domains are known to be sensitive to ageing; pattern recognition memory (PRM, mean latency of a correct response over 24 trials), DMS (mean correct latency over 40 trials), PAL (number of errors made), spatial span (longest sequence remembered correctly), spatial working memory (number of ‘between’ errors made), SRT (mean of 8 correct trials) and five-choice reaction time (RTI, mean of 8 correct trials). Exploratory factor analysis of a 10-year change in the seven scores was conducted using a latent difference approach, adjusting for baseline cognitive performance by fitting the baseline test result as a linear covariate. This measure indicated a 10-year change in score assuming all individuals started at the same baseline performance. It is mathematically the same as fitting the baseline as a covariate with the follow-up measurement as the outcome variable. The first extracted factor, which explained 25% of the variance and was strongly associated with age (standardised beta −0.066, *p* < 0.001), was considered the ARC score [25]. Factor loadings showed that the greatest loadings for the ARC score were tests with a speed component (PRM (0.60), RTI (0.59) and DMS (0.58)) [25]. A positive change in the score indicated an improvement in cognition over 10 years. In sensitivity analyses, we examined these longitudinal results with the change in fruit and vegetable intake, baseline systolic blood pressure and change in caffeine intake added to the models, in addition to the covariates listed below.

Second, in cross-sectional analysis, participants were ranked into tertiles of intake for flavonoid subclasses and we examined associations with test scores assessing cognitive domains known to be sensitive to changes in flavonoid intake (PAL, DMS, IED, SRT). We chose not to include all the cognitive domains incorporated in our primary longitudinal analysis into the cross-sectional analysis in order to reduce multiple testing.

In both the longitudinal and cross-sectional analyses, we used all participants and treated twins as individuals (individual-level analysis). The effect of this clustered sample design on the standard errors was accounted for by calculating robust standard errors. Associations with cognition variables were assessed with the use of ANCOVA for both intakes of flavonoid subclasses. All models were adjusted for age (years), BMI (kg/m^2^), current smoking (yes or no), physical activity (active, moderately active, inactive), post-menopausal status (yes or no), vitamin supplement use (yes or no), occupation (professional, intermediate, skilled non-manual, skilled manual, partly skilled or unskilled), a highest education level (no qualifications; O-Level, GCSE, NVQ2/SVQ2, or Scottish Intermediate; Scottish Higher, NVQ3, city and guilds, Pitman, A Level, Scottish Advanced Higher, or higher vocational training; university degree, postgraduate degree, NVQ5, or SVQ5), verbal IQ score (NART score), presence of learning disabilities, depression or neurological conditions (yes or no), and intakes of energy (kcal/d, in tertiles), alcohol (g/d, in tertiles) and fat (g/d, in tertiles). In the longitudinal analyses, models were adjusted for a 10-year change in the covariates. We performed multiple imputations of missing covariate data using multivariate imputation by chained equations with 15 imputations (equal to the maximum percentage of incomplete cases). The imputation model included all variables from the analysis models. We checked for effect modification by including interaction terms for age group (<40 years, 40–59 years and ≥ 60 years) and flavonoid intake in the models.

Thirdly, taking the statistical approach widely used in matched case-control studies, we examined monozygotic twin pairs who were discordant for baseline intakes of the different flavonoid subclasses that were significantly associated with cognition in the previous analyses. Discordance was defined as a within-pair difference in intake of >1 SD. We assigned each twin within a discordant pair to higher or lower baseline intake for each subclass, and with the use of paired-sample *t* tests examined whether cognitive variables differed between the twin with higher intake and that of the co-twin with lower intake. We determined differences between the higher and lower intake twins for all the covariates included in the main analyses with the use of paired sample *t* tests or McNemar chi-squared test.

In a further co-twin analysis in sub-set analysis of 32 monozygotic twins with baseline dietary data and MRI-neuroimaging data after 12 years, we compared volumes of the hippocampus and parahippocampus between the higher and lower intake twin within each pair (*n* = 16) after adjustment for a limited set of covariates: age (years) and highest education level category using ANCOVA.

*P* <0.05 was considered statistically significant for all these hypothesis-driven exploratory analyses. Statistical analyses were performed with Stata statistical software (version 16; StataCorp LP), and syntax is available on request to the authors.

## Results

Characteristics and dietary intakes of the 1126 female participants, aged 18–89 years, are shown in Table 1; 48% of subjects (*n* = 542) were monozygotic twins. The mean total flavonoid intake was 1.1 g/d (SD 0.6), and in the longitudinal dataset mean, a 10-year change was −30.6 mg/d (SD 472). Of the participants in the longitudinal dataset (*n* = 224), 70% were aged 40–59 and 30% aged over 60 years at baseline (data not shown). The participant characteristics according to tertile of baseline flavonoid subclass intakes and change in flavonoid intakes are presented in Supplemental Tables 1 and 2, respectively. In comparison to all recruited participants (*n* = 5772), the current sample was older (56.5 years vs 49.7 years, *p*<0.01) and contained more monozygotic twins (48% vs 41%, *p*<0.01). There were no significant differences in BMI between the groups (25.3 kg/m^2^ vs 25.4 kg/m^2^, *p* = 0.83).

**Table 1:** Characteristics and dietary intakes of the whole sample of female twins (*n* = 1126) and the longitudinal subset (*n* = 224) at baseline and over 10 years

Characteristic	Whole sample		Longitudinal subset	
	***n***=	Value	***n***=	Value
Age, years	1126	56.5 (12.9)	224	54.9 (7.7)
Monozygotic, yes, % (*n*)	1126	48.1 (542)	224	34.8 (78)
BMI, kg/m^2^	1126	25.3 (4.5)	224	25.3 (4.2)
Current smoker, yes, % (*n*)	1121	8.1 (91)	224	10.3 (23)
Physically active, yes, % (*n*)	1119	25.7 (288)	224	25.9 (58)
Post-menopausal, yes, % (*n*)	1126	71.4 (804)	224	61.2 (137)
Highest education, no qualifications, % (*n*)	1126	11.6 (131)	224	18.8 (42)
Occupation, professional, % (*n*)	956	37.2 (356)	224	46.4 (104)
NART, verbal IQ score	1104	116 (7.2)	222	115 (7.1)
Total flavonoids, mg/day	1126	1107 (621)	224	1195 (591)
Flavanones, mg/day	1126	29.1 (31.5)	224	34.6 (30.3)
Anthocyanins, mg/day	1126	21.0 (17.7)	224	21.4 (17.1)
Flavan-3-ols, mg/day	1126	244 (179)	224	233 (132)
Flavonols, mg/day	1126	44.4 (20.5)	224	47.6 (19.3)
Flavones, mg/day	1126	2.2 (1.5)	224	2.3 (1.4)
Polymers, mg/day	1126	766 (458)	224	856 (439)
Proanthocyanidins, mg/day	1126	255 (122)	224	286 (116)
Total flavonoids, a 10-year change	–	–	224	−30.6 (472)
Flavanones, a 10-year change	–	–	224	−3.1 (36.7)
Anthocyanins, a 10-year change	–	–	224	4.1 (21.3)
Flavan-3-ols, a 10-year change	–	–	224	17.7 (137)
Flavonols, a 10-year change	–	–	224	−0.9 (16.0)
Flavones, a 10-year change	–	–	224	0.2 (1.7)
Polymers, a 10-year change	–	–	224	−48.8 (337)
Proanthocyanidins, a 10-year change	–	–	224	−0.2 (139)
Alcohol, g/day	1126	8.9 (12.3)	224	10.2 (12.4)
Fat, g/day	1126	66.7 (24.9)	224	67.9 (22.5)
Energy, kcal/day	1126	1875 (547)	224	1974 (470)
Vitamin supplement use, yes, % (*n*)	1092	54.0 (590)	209	64.6 (135)
Paired-associate learning, stages	1126	7.7 (0.67)	–	–
Delayed matching to sample, number correct	541	19.5 (6.1)	–	–
Simple reaction time, ms	649	351 (77.0)	–	–
Intra-extra dimensional set shift, stages	667	8.2 (1.5)	–	–
Age-related cognitive score, a 10-year change score	–	–	224	0.03 (0.93)

Values are mean (SD) or % (*n*) where indicated and refer to baseline unless a 10-year change is indicated

### Longitudinal analysis: a 10-year change in intake of flavonoid subclasses and a 10-year change in age-related cognition score

**Fig. 1:**
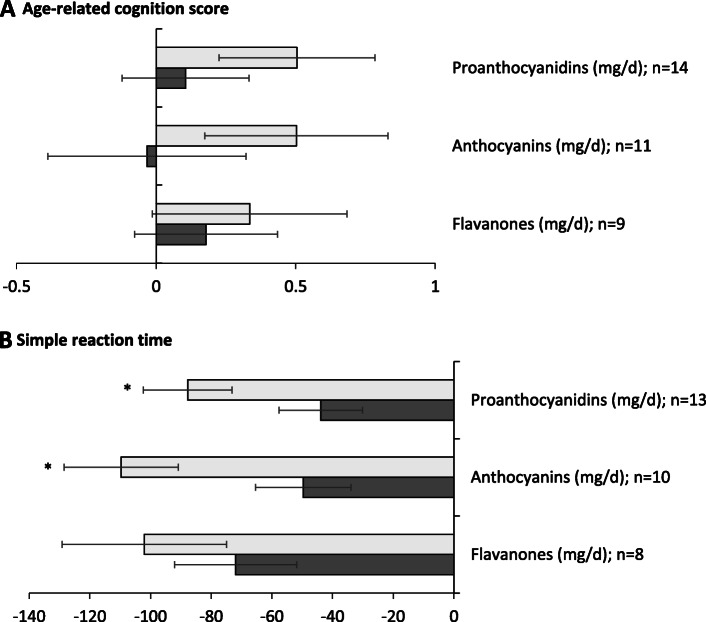
A 10-year change in age-related cognitive score by tertiles of a 10-year change in flavonoid subclass intake. Values are adjusted means (95% CI), *n* = 224. Means were adjusted for a 10-year change in age (years), BMI (kg/m^2^), current smoking (yes or no), physical activity (active, moderately active, inactive), post-menopausal status (yes or no), vitamin supplement use (yes or no) and intakes of energy (kcal/d), alcohol (g/d) and fat (g/d), occupation (professional, intermediate, skilled non-manual, skilled manual, partly skilled or unskilled), highest education level (no qualifications; O-Level, GCSE, NVQ2/SVQ2 or Scottish Intermediate; Scottish Higher, NVQ3, city and guilds, Pitman, A Level, Scottish Advanced Higher, or higher vocational training; university degree, postgraduate degree, NVQ5 or SVQ5), verbal IQ score (NART score) and the presence of learning disabilities, depression or neurological conditions (yes or no). *P-trend <0.05, calculated using ANCOVA. A positive change in cognitive score translates as an improvement over 10 years and a negative score is a decline

In the longitudinal dataset, the greatest increase in habitual intakes of flavanones (T3-T1 0.45, 95% CI 0.13, 0.77; *p* = 0.01) and anthocyanins (T3-T1 0.45, 95% CI 0.08, 0.81; *p* = 0.02) over 10 years was associated with significantly greater improvement in ARC score (a positive change in ARC score) when compared to the lowest change in intake (Fig. 1 and Supplemental Table 3). We did not observe any longitudinal change in cognition related to change in intakes of the other flavonoid subclasses.

In sensitivity analysis for our primary analysis, we examined the impact of adding additional covariates to the multivariate model for change in ARC score. The results were not markedly changed when adding change in total fruit and vegetable intake (flavanones 0.42 (95% CI 0.06, 0.78; *p* = 0.02) and anthocyanins 0.40 (95% CI 0.04, 0.77; *p* = 0.04)), baseline systolic blood pressure (flavanones 0.45 (95% CI 0.13, 0.77; *p* = 0.01) and anthocyanins 0.45 (95% CI 0.08, 0.81; *p* = 0.04)) or change in caffeine intake (flavanones 0.44 (95% CI 0.07, 0.81; *p* = 0.01) and anthocyanins 0.44 (95% CI 0.07, 0.81; *p* = 0.03)) (data not shown).

### Cross-sectional analysis: associations between intake of flavonoid subclasses and scores for relevant cognitive domains

In cross-sectional analysis, a higher intake of flavanones (T3-T1 0.12, 95% CI 0.02, 0.21; *p* = 0.02, Table 2) and proanthocyanidins (T3-T1 0.13, 95% CI 0.02, 0.24; *p* = 0.02) was associated with a greater number of PAL stages completed. A higher intake of anthocyanins was significantly associated with higher IED stages completed (T3-T1 −0.52, 95% CI 0.19, 0.84; *p* = 0.001) and with faster simple reaction times (T3-T1 −18.1, 95% CI −35.4, −0.7; *p* = 0.04). No associations were observed for the other subclasses. For the significant associations observed, there were no significant interactions between age group and flavonoid subclass intake.

**Table 2:** Measures of cognition by tertile of flavonoid subclass intake in 1126 females aged 18–89 years

Cognition variable	***n***=	Subclass, mg/day	T1	T2	T3	***P***=
			Mean (95% CI)	Mean (95% CI)	Mean (95% CI)	
PAL, stages completed	1126	Total flavonoids	7.71 (7.62, 7.80)	7.74 (7.68, 7.81)	7.77 (7.71, 7.82)	0.22
		Flavanones	7.69 (7.61, 7.76)	7.73 (7.67, 7.80)	7.80 (7.74, 7.87)	0.02
		Anthocyanins	7.70 (7.62, 7.78)	7.78 (7.72, 7.84)	7.74 (7.66, 7.82)	0.44
		Flavan-3-ols	7.70 (7.61, 7.78)	7.74 (7.67, 7.81)	7.79 (7.73, 7.84)	0.06
		Flavonols	7.69 (7.61, 7.78)	7.76 (7.69, 7.83)	7.77 (7.71, 7.83)	0.12
		Flavones	7.76 (7.69, 7.82)	7.72 (7.66, 7.78)	7.75 (7.66, 7.83)	0.81
		Polymers	7.71 (7.62, 7.79)	7.74 (7.68, 7.81)	7.77 (7.71, 7.83)	0.21
		Proanthocyanidins	7.65 (7.56, 7.74)	7.79 (7.74, 7.84)	7.78 (7.72, 7.85)	0.01
IED, stages completed	667	Total flavonoids	8.2 (8.0,8.5)	8.2 (8.0,8.4)	8.1 (7.9,8.3)	0.39
		Flavanones	8.2 (8.0,8.4)	8.1 (7.9,8.3)	8.2 (8.0,8.4)	0.81
		Anthocyanins	7.8 (7.6,8.1)	8.3 (8.1,8.5)	8.4 (8.2,8.5)	<0.001
		Flavan-3-ols	8.2 (8.0,8.4)	8.2 (8.0,8.4)	8.1 (7.9,8.3)	0.28
		Flavonols	8.3 (8.0,8.5)	8.1 (7.8,8.3)	8.2 (8.0,8.4)	0.58
		Flavones	8.1 (7.9,8.3)	8.2 (8.0,8.4)	8.2 (8.0,8.4)	0.46
		Polymers	8.2 (8.0,8.4)	8.2 (8.0,8.4)	8.1 (7.9,8.3)	0.40
		Proanthocyanidins	8.2 (8.0,8.4)	8.1 (7.9,8.3)	8.2 (8.0,8.4)	0.88
SRT, *milliseconds*	649	Total flavonoids	350 (339,360)	348 (338,359)	356 (346,365)	0.38
		Flavanones	349 (337,361)	354 (342,365)	351 (341,361)	0.83
		Anthocyanins	361 (349,373)	350 (341,360)	342 (332,353)	0.04
		Flavan-3-ols	349 (339,359)	350 (339,360)	355 (345,365)	0.42
		Flavonols	352 (341,363)	346 (336,357)	355 (346,365)	0.60
		Flavones	361 (348,374)	349 (339,359)	344 (334,353)	0.05
		Polymers	350 (340,360)	347 (336,357)	357 (347,367)	0.32
		Proanthocyanidins	356 (343,368)	349 (339,359)	349 (339,358)	0.37
DMS, total correct	541	Total flavonoids	20.0 (18.9,21.0)	19.5 (18.4,20.5)	19.0 (18.1,19.9)	0.11
		Flavanones	19.9 (18.8,21.0)	19.6 (18.7,20.5)	19.0 (18.1,19.9)	0.20
		Anthocyanins	19.1 (18.2,20.0)	19.7 (18.7,20.7)	19.6 (18.5,20.7)	0.48
		Flavan-3-ols	19.9 (18.9,20.9)	19.4 (18.4,20.3)	19.2 (18.2,20.1)	0.26
		Flavonols	20.0 (18.9,21.0)	19.6 (18.6,20.5)	18.9 (18.0,19.8)	0.09
		Flavones	19.7 (18.7,20.7)	19.6 (18.6,20.6)	19.1 (18.1,20.2)	0.45
		Polymers	20.1 (19.0,21.1)	19.4 (18.4,20.4)	19.0 (18.1,19.9)	0.08
		Proanthocyanidins	20.1 (19.0,21.2)	19.2 (18.2,20.1)	19.2 (18.3,20.1)	0.19

Values are adjusted means (95% CI), *n* =1126. Means were adjusted for age (years), BMI (kg/m^2^), current smoking (yes or no), physical activity (active, moderately active, inactive), post-menopausal status (yes or no), vitamin supplement use (yes or no), occupation (professional, intermediate, skilled non-manual, skilled manual, partly skilled or unskilled), highest education level (no qualifications; O-Level, GCSE, NVQ2/SVQ2 or Scottish Intermediate; Scottish Higher, NVQ3, city and guilds, Pitman, A Level, Scottish Advanced Higher or higher vocational training; university degree, postgraduate degree, NVQ5 or SVQ5), verbal IQ score (NART score), presence of learning disabilities, depression or neurological conditions (yes or no), and intakes of energy (kcal/d, in tertiles), alcohol (g/d, in tertiles) and fat (g/d, in tertiles). *P* P-trend calculated using ANCOVA. *DMS* delayed matching to samples, *IED* intra-extra dimensional set shift, *PAL* paired-associate learning, *SRT* simple reaction time

### Monozygotic co-twin analysis: comparison of a 10-year change in cognitive scores in twin pairs discordant for intake of flavonoid subclasses and medial temporal lobe volumes

**Fig. 2:**
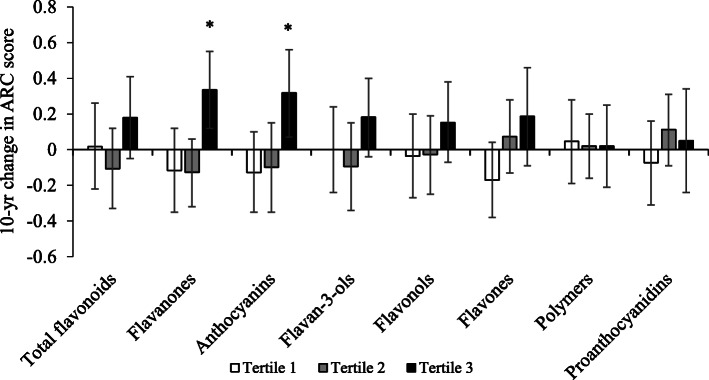
A 10-year change in cognitive measures in monozygotic co-twins discordant for flavonoid subclass intake. Bars represent the mean and error bars of the standard error of a 10-year change in ARC score (panel **A**) and a 10-year change in simple reaction time (panel **B**) in the high (white bars) and low intake (black bars) twins. *n* = is the number of pairs included in each analysis. Discordance was defined as a within-pair difference in intake ≥1 SD. *p values <0.05 calculated using paired sample *t* tests for comparison of twins with higher intake with twins with lower intake. Measures of delayed matching to samples, intra-extra dimensional set shift and paired-associate learning were non-significant and are not shown. In the ARC analysis, two twin pairs were discordant for all three subclasses; two twin pairs were discordant for anthocyanins and flavanones, three twin pairs were discordant for anthocyanins and proanthocyanidins and one twin pair was discordant for proanthocyanidins and flavanones

Intakes between discordant monozygotic twin pairs differed by 89.6 mg/d for flavanones, 26.1 mg/d for anthocyanins and 224 mg/d for proanthocyanidins. Within each twin pair, the twin with higher intakes of anthocyanins (*p*<0.01) and proanthocyanidins (*p* = 0.01) had improved reaction times after 10 years (Fig. 2). There were no significant differences between the discordant pairs for the covariates (BMI, current smoking, physical activity, post-menopausal status, vitamin supplement use, occupation, highest education level, verbal IQ score, presence of learning disabilities, depression or neurological conditions, and intakes of alcohol and fat), with the exception of energy intake where a significant difference was observed in the twins discordant for flavanone intake (451.3 kcal/d, *p*<0.01).

In sub-set analysis of 16 monozygotic twin pairs where we had MRI data (mean age 52.4 years, SD 8.4), we found baseline intakes of anthocyanins and proanthocyanidins to be associated with hippocampal and parahippocampal volumes after 12 years. Higher intakes of anthocyanins were associated with higher left hippocampal (2.0% comparing the higher and lower intake twins, *p* = 0.01) and parahippocampal volumes (2.3% comparing the higher and lower intake twins, *p* = 0.03) and higher intakes of proanthocyanidins were associated with higher left hippocampal volumes (2.0% comparing the higher and lower intake twins, *p* = 0.01, *p* = 0.02) (Table 3).

**Table 3:** Regional brain volumes stratified by high and low flavonoid subclass intake in 32 female twins aged 42–69 years

Brain regions	Subclass, mg/day	Low intake twins (***n***=16)	High intake twins (***n***=16)	***P***=
		Mean (95% CI)	Mean (95% CI)	
Left hippocampus standardised volumes	Flavanones	0.432 (0.421,0.443)	0.433 (0.418,0.447)	0.87
	Anthocyanins	0.428 (0.417,0.440)	0.437 (0.423,0.450)	0.01
	Proanthocyanidins	0.428 (0.416,0.440)	0.437 (0.424,0.450)	0.02
Right hippocampus standardised volumes	Flavanones	0.407 (0.395,0.419)	0.408 (0.395,0.422)	0.76
	Anthocyanins	0.408 (0.394,0.421)	0.408 (0.396,0.420)	0.90
	Proanthocyanidins	0.404 (0.391,0.418)	0.411 (0.400,0.422)	0.06
Left para-hippocampus standardised volumes	Flavanones	0.434 (0.424,0.443)	0.429 (0.417,0.441)	0.33
	Anthocyanins	0.426 (0.417,0.436)	0.436 (0.425,0.447)	0.03
	Proanthocyanidins	0.428 (0.418,0.438)	0.435 (0.423,0.446)	0.18
Right para-hippocampus standardised volumes	Flavanones	0.470 (0.458,0.481)	0.461 (0.448,0.474)	0.07
	Anthocyanins	0.467 (0.453,0.480)	0.464 (0.453,0.475)	0.61
	Proanthocyanidins	0.466 (0.454,0.478)	0.464 (0.451,0.477)	0.72

Values are adjusted means (95% CI), *n* = 32. Means were adjusted for age (years) and highest education level (no qualifications; O-Level, GCSE, NVQ2/SVQ2, or Scottish Intermediate; Scottish Higher, NVQ3, city and guilds, Pitman, A Level, Scottish Advanced Higher or higher vocational training; university degree, postgraduate degree, NVQ5 or SVQ5). Volumes are standardised for intracranial volume and brain folding during processing

## Discussion

To our knowledge, this is the first study to integrate longitudinal, cross-sectional, co-twin and mechanistic data to examine the associations between habitual intake of flavonoid sub-classes and cognition. Higher habitual intakes of anthocyanins, flavanones and proanthocyanidins were associated with improvements in measures of cognition and larger brain volumes across the three sets of data analyses (longitudinal, cross-sectional and co-twin) with particular evidence for speed of processing and episodic memory but not other memory domains. Specifically, these novel data suggest that small increases in intakes of flavanones (64.9 mg/day, SE 4.2) and anthocyanins (37.4 mg/day, SE 2.5) were associated with an improvement in ARC score over 10 years. The change in ARC score was 3.9 and 3.5 times greater in the highest consumers of flavanones and anthocyanins compared to the lowest consumers, respectively, and equivalent to approximately 4 years of ageing (calculated by dividing the β coefficients per quintile of flavanone and anthocyanin intake (0.22 and 0.21, respectively) by the coefficient per year of age (−0.05 for both sub-classes). These results related to a mean difference in intake of 64.9 mg/d of flavanones, equivalent to around 2 medium oranges (342 g/day) and 37.4 mg/day anthocyanins (0.2 cup/d blueberries (23 g/day) or around 1 cup/day of strawberries (138 g/day). Thereby providing evidence that effects on cognition can be obtained by achievable and simple dietary changes to the habitual diet.

It was a particular strength of these analyses that flavonoid subclass intakes were still predictive within twin pairs and suggest that small changes in habitual intake of flavonoid-rich foods have the potential to attenuate cognitive ageing. The results of our primary analyses were not markedly changed after the addition of total fruit and vegetable intake to the models suggesting that the observed effects were independent of change in overall intakes of fruit and vegetables and specific to change in particular flavonoid-rich foods, adding further weight to the potential importance of dietary flavonoids.

Our cross-sectional analysis suggested that higher intakes of anthocyanins were significantly associated with improved measures of executive function and psychomotor function and proanthocyanidins with episodic memory. These findings support those of a short-term (90 days) randomised controlled trial where daily intake of blueberries (equivalent to 19.2 mg/day anthocyanins) improved episodic memory and executive function test scores [35]. They also corroborate findings from a 6-month intervention in 122 older adults with a wild blueberry extract (containing 14 mg/day total anthocyanins) which was found to facilitate better episodic memory performance and improve cardiovascular function, but not executive function [36]. In our cross-sectional analyses, the difference in anthocyanin intake associated with our findings was 31.7 mg/d (SE 0.86) equivalent to 19 g blueberries (< 1/2 cup) [37], supporting results from these RCTs that intake of anthocyanins and can improve cognitive function even at relatively low habitual intakes.

It is well established that flavonoid sub-classes, especially anthocyanins, are associated with improved cardiovascular function, including evidence from this cohort where the higher intake of anthocyanins was associated with lower central blood pressure and arterial stiffness [31]. In sensitivity analyses, we added systolic blood pressure as a covariate to our analysis and found no material change to the results. We also showed in subset analyses that intakes of anthocyanins and proanthocyanidins were associated with increased hippocampal volumes. This suggests the mechanisms underlying the beneficial effects of dietary flavonoids on cognitive health are not exclusively vascular-related and brain-specific mechanisms are also relevant. This is supported by evidence from animal studies showing that anthocyanin and proanthocyanidin metabolites can pass the blood-brain barrier [7]. Anthocyanin metabolites were found in the hippocampus and cortex, brain regions known to be important for learning and memory, of rats fed a blueberry supplemented diet for 10 weeks, whilst no metabolites were detected in control-diet fed rats [19]. Interventions with grape seed extracts and grape juice, rich in anthocyanins and proanthocyanidins, were found to reduce amyloid content and plaque burden in Alzheimer’s disease transgenic mice compared to control; furthermore, polyphenol metabolites from the grape seed extracts and grape juice interventions significantly improved acute oligomeric amyloid-β peptide-induced long-term potentiation deficits in hippocampal slices, one of the major cellular mechanisms underlying synaptic plasticity [38].

In our MRI sub-study, we found greater left hippocampus volumes associated with higher intakes of anthocyanins and proanthocyanidins. Previous studies have shown diet-hippocampus volume associations are stronger in the left than in the right hippocampus [39]. The human hippocampus is key in the formation of episodic memory, with the left hippocampus predominantly associated with the storage of verbal and narrative memory [40]. This corroborates our findings of an association between anthocyanin and proanthocyanidin intakes and visuospatial episodic memory, measured using the PAL test. As the deterioration of the hippocampus leads to memory impairment, and increased permeability of the blood-brain barrier is often most pronounced in the hippocampus [41], low-cost, accessible preventions and treatments for reducing hippocampal tissue loss are therefore a public health priority. This study adds evidence to support the role of dietary intervention with anthocyanins and proanthocyanidins as a means to improve cognitive health.

Strengths of the current study include the integration of longitudinal, cross-sectional and co-twin data which increases the robustness of our findings and reduces confounding and reverse causation, the large sample of well-characterised participants, the measurement of all major flavonoid sub-classes and the repeated measures of diet and cognition over 10 years. Change-on-change analysis allowed us to control for within-person variation and present less biassed associations and our use of a co-twin case-control model allowed us to examine associations independently of genetic confounding [42, 43]. The FFQ used in the current study captured the main sources of flavonoids present in the habitual diet has been shown to reflect habitual dietary intake and has the ability to rank participants according to intake of flavonoid-rich foods [44]. Furthermore, self-reported fruit and vegetable intakes correlate well with urinary flavonoid concentrations [45]. There is however a likely underestimation of real flavonoid intake due to omission of food items in the FFQ, unknown food composition data and variability in the flavonoid content of foods due to geographical origin, season and processing methods. We partly overcame these limitations by ranking participants according to intake rather than relying on absolute intake and applying a correction factor to seasonal fruits and vegetables. Further limitations include the relatively small sample size and the use of cross-sectional analyses for some of these results, which meant we are unable to infer causation from these particular findings. Due to the novel and exploratory nature of the analyses, a number of hypothesis-driven comparisons were made and multiple testing was not accounted for. Furthermore, residual confounding is possible despite our detailed adjustment for a range of dietary and lifestyle covariates. Finally, previous research has reported differences in flavonoid intake between males and females [46], and as our cohort consisted only of females, we cannot extrapolate our results to males.

## Conclusions

In conclusion, our longitudinal data in female twins suggest that several flavonoids, specifically the flavanone and anthocyanin sub-classes attenuate cognitive ageing and further co-twin analyses demonstrated associations independently of shared genetic and common environmental factors. These findings were independent of total fruit and vegetable intake and found with dietary achievable intakes, making them relevant for public health recommendations to improve cognition. Our results, particularly those for hippocampal volume, need to be confirmed in larger studies and randomised controlled trials that include both males and females.

## Supplementary Information

**Additional file 1: Table S1.** Baseline characteristics by tertile of flavonoid subclass intake in 1126 females. **Table S2.** Baseline characteristics by tertile of 10-year change in flavonoid subclass intake in 224 females. **Table S3.** 10-year change in age-related cognitive score by tertiles of 10-year change in flavonoid subclass intake. **Figure S1.** Participant flow chart.

## Data Availability

The datasets used and analysed during the current study are available from the corresponding author on reasonable request.
