# Supplementary material for: Increased habitual flavonoid intake predicts attenuation of cognitive ageing in twins
Source: BMC Med. 2021 Aug 23;19:185. doi: 10.1186/s12916-021-02057-7 (PMC8381583; doi:10.1186/s12916-021-02057-7)
Supplement: Supplementary file 1 — Additional file 1: Table S1. Baseline characteristics by tertile of flavonoid subclass intake in 1126 females. Table S2. Baseline characteristics by tertile of 10-year change in flavonoid subclass intake in 224 females. Table S3. 10-year change in age-related cognitive score by tertiles of 10-year change in flavonoid subclass intake. Figure S1. Participant flow chart. [file 12916_2021_2057_MOESM1_ESM.docx]

**Table S1: Baseline characteristics by tertile of flavonoid subclass intake in 1126 females**

| **Subclass, mg/d** | **Characteristic** | **n=** | **T1** | **T2** | **T3** | **P=** |
| --- | --- | --- | --- | --- | --- | --- |
| Total flavonoids | Age, years | 1126 | 54.3 (14.0) | 58.3 (12.2) | 56.9 (12.2) | 0.01 |
|  | BMI, kg/m^2^ | 1126 | 25.5 (5.0) | 25.0 (4.1) | 25.4 (4.5) | 0.84 |
|  | Current smoker, yes, % (n) | 1121 | 43 (3.8) | 22 (2.0) | 26 (2.3) | 0.01 |
|  | Physically active, yes, % (n) | 1119 | 124 (11.1) | 116 (10.4) | 119 (10.6) | 0.10 |
|  | Supplement use, yes, % (n) | 1092 | 193 (17.7) | 209 (19.1) | 188 (17.2) | 0.32 |
|  | Post-menopausal, yes, % (n) | 1126 | 246 (21.8) | 284 (25.2) | 274 (24.3) | 0.01 |
|  | Highest education, none, % (n) | 1126 | 42 (3.7) | 37 (3.3) | 52 (4.6) | 0.11 |
|  | Occupation, professional, % (n) | 956 | 105 (11.0) | 118 (12.3) | 133 (13.9) | 0.46 |
| Flavanones | Age, years | 1126 | 56.1 (13.3) | 56.6 (12.8) | 56.8 (12.7) | 0.50 |
|  | BMI, kg/m^2^ | 1126 | 25.5 (4.8) | 25.2 (4.4) | 25.3 (4.4) | 0.44 |
|  | Current smoker, yes, % (n) | 1121 | 39 (3.5) | 31 (2.8) | 21 (1.9) | 0.05 |
|  | Physically active, yes, % (n) | 1119 | 139 (12.4) | 121 (10.8) | 99 (8.8) | 0.03 |
|  | Supplement use, yes, % (n) | 1092 | 189 (17.3) | 191 (17.5) | 210 (19.2) | 0.15 |
|  | Post-menopausal, yes, % (n) | 1126 | 268 (23.8) | 262 (23.3) | 274 (24.3) | 0.62 |
|  | Highest education, none, % (n) | 1126 | 50 (4.4) | 47 (4.2) | 34 (3.0) | 0.09 |
|  | Occupation, professional, % (n) | 956 | 106 (11.1) | 120 (12.6) | 130 (13.6) | <0.001 |
| Anthocyanins | Age, years | 1126 | 53.7 (14.3) | 56.5 (12.1) | 59.4 (11.7) | <0.001 |
|  | BMI, kg/m^2^ | 1126 | 25.3 (4.6) | 25.4 (4.6) | 25.3 (4.4) | 0.92 |
|  | Current smoker, yes, % (n) | 1121 | 41 (3.7) | 24 (2.1) | 26 (2.3) | 0.04 |
|  | Physically inactive, yes, % (n) | 1119 | 140 (12.5) | 118 (10.5) | 101 (9.0) | 0.04 |
|  | Supplement use, yes, % (n) | 1092 | 183 (16.8) | 197 (18.0) | 210 (19.2) | 0.05 |
|  | Post-menopausal, yes, % (n) | 1126 | 250 (22.2) | 266 (23.6) | 288 (25.6) | 0.01 |
|  | Highest education, none, % (n) | 1126 | 46 (4.1) | 40 (3.6) | 45 (4.0) | 0.07 |
|  | Occupation, professional, % (n) | 956 | 114 (11.9) | 124 (13.0) | 118 (12.3) | 0.10 |
| Flavan-3-ols | Age, years | 1126 | 53.9 (14.2) | 58.4 (12.3) | 57.2 (11.8) | <0.001 |
|  | BMI, kg/m^2^ | 1126 | 25.6 (5.0) | 25.1 (4.0) | 25.3 (4.5) | 0.46 |
|  | Current smoker, yes, % (n) | 1121 | 47 (4.2) | 19 (1.7) | 25 (2.2) | <0.001 |
|  | Physically active, yes, % (n) | 1119 | 124 (11.1) | 124 (11.1) | 111 (9.9) | 0.05 |
|  | Supplement use, yes, % (n) | 1092 | 189 (17.3) | 204 (18.7) | 197 (18.0) | 0.43 |
|  | Post-menopausal, yes, % (n) | 1126 | 238 (21.1) | 288 (25.6) | 278 (24.7) | <0.001 |
|  | Highest education, none, % (n) | 1126 | 44 (3.9) | 39 (3.5) | 48 (4.3) | 0.48 |
|  | Occupation, professional, % (n) | 956 | 110 (11.5) | 118 (12.3) | 128 (13.4) | 0.59 |
| Flavonols | Age, years | 1126 | 54.9 (13.8) | 57.9 (12.5) | 56.7 (12.2) | 0.07 |
|  | BMI, kg/m^2^ | 1126 | 25.6 (5.0) | 25.1 (4.0) | 25.3 (4.5) | 0.51 |
|  | Current smoker, yes, % (n) | 1121 | 44 (3.9) | 20 (1.8) | 27 (2.4) | <0.001 |
|  | Physically active, yes, % (n) | 1119 | 122 (10.9) | 123 (11.0) | 114 (10.2) | 0.26 |
|  | Supplement use, yes, % (n) | 1092 | 198 (18.1) | 194 (17.8) | 198 (18.1) | 0.84 |
|  | Post-menopausal, yes, % (n) | 1126 | 249 (22.1) | 280 (24.9) | 275 (24.4) | 0.02 |
|  | Highest education, none, % (n) | 1126 | 41 (3.6) | 47 (4.2) | 43 (3.8) | 0.06 |
|  | Occupation, professional, % (n) | 956 | 101 (10.6) | 122 (12.8) | 133 (13.9) | 0.31 |
| Flavones | Age, years | 1126 | 56.2 (13.5) | 56.3 (13.2) | 57.1 (12.0) | 0.36 |
|  | BMI, kg/m^2^ | 1126 | 25.5 (4.7) | 25.4 (4.3) | 25.2 (4.6) | 0.37 |
|  | Current smoker, yes, % (n) | 1121 | 38 (3.4) | 29 (2.6) | 24 (2.1) | 0.15 |
|  | Physically active, yes, % (n) | 1119 | 143 (12.8) | 109 (9.7) | 107 (9.6) | 0.02 |
|  | Supplement use, yes, % (n) | 1092 | 182 (16.7) | 188 (17.2) | 220 (20.1) | 0.01 |
|  | Post-menopausal, yes, % (n) | 1126 | 264 (23.4) | 264 (23.4) | 276 (24.5) | 0.51 |
|  | Highest education, none, % (n) | 1126 | 63 (5.6) | 34 (3.0) | 34 (3.0) | <0.001 |
|  | Occupation, professional, % (n) | 956 | 124 (13.0) | 124 (13.0) | 108 (11.3) | 0.16 |
| Polymers | Age, years | 1126 | 54.7 (13.8) | 58.0 (12.5) | 56.9 (12.2) | 0.02 |
|  | BMI, kg/m^2^ | 1126 | 25.4 (4.9) | 25.1 (4.2) | 25.4 (4.5) | 1.00 |
|  | Current smoker, yes, % (n) | 1121 | 42 (3.7) | 23 (2.1) | 26 (2.3) | 0.02 |
|  | Physically active, yes, % (n) | 1119 | 120 (10.7) | 120 (10.7) | 119 (10.6) | 0.14 |
|  | Supplement use, yes, % (n) | 1092 | 199 (18.2) | 204 (18.7) | 187 (17.1) | 0.57 |
|  | Post-menopausal, yes, % (n) | 1126 | 249 (22.1) | 281 (25.0) | 274 (24.3) | 0.02 |
|  | Highest education, none, % (n) | 1126 | 40 (3.6) | 38 (3.4) | 53 (4.7) | 0.14 |
|  | Occupation, professional, % (n) | 956 | 102 (10.7) | 119 (12.4) | 135 (14.1) | 0.35 |
| Proanthocyanidins | Age, years | 1126 | 55.1 (14.0) | 56.9 (12.5) | 57.5 (12.1) | 0.01 |
|  | BMI, kg/m^2^ | 1126 | 25.3 (4.5) | 25.3 (4.8) | 25.4 (4.3) | 0.87 |
|  | Current smoker, yes, % (n) | 1121 | 37 (3.3) | 33 (2.9) | 21 (1.9) | 0.08 |
|  | Physically active, yes, % (n) | 1119 | 132 (11.8) | 115 (10.3) | 112 (10.0) | 0.13 |
|  | Supplement use, yes, % (n) | 1092 | 186 (17.0) | 192 (17.6) | 212 (19.4) | 0.03 |
|  | Post-menopausal, yes, % (n) | 1126 | 263 (23.4) | 267 (23.7) | 274 (24.3) | 0.64 |
|  | Highest education, none, % (n) | 1126 | 43 (3.8) | 50 (4.4) | 38 (3.4) | 0.19 |
|  | Occupation, professional, % (n) | 956 | 97 (10.1) | 128 (13.4) | 131 (13.7) | 0.04 |

Values are mean (SD) or % (n) where indicated.

**Table S2: Baseline characteristics by tertile of 10-year change in flavonoid subclass intake in 224 females**

| **Subclass, mg/d** | **Characteristic** | **n=** | **T1** | **T2** | **T3** | **P=** |
| --- | --- | --- | --- | --- | --- | --- |
| Total flavonoids | Age, years | 224 | 55.8 (8.0) | 55.5 (7.9) | 53.5 (6.9) | 0.07 |
|  | BMI, kg/m^2^ | 224 | 25.4 (4.2) | 25.6 (4.4) | 24.8 (4.0) | 0.39 |
|  | Current smoker, yes, % (n) | 224 | 8 (3.6) | 7 (3.1) | 8 (3.6) | 0.95 |
|  | Physically active, yes, % (n) | 224 | 13 (5.8) | 11 (4.9) | 12 (5.4) | 0.96 |
|  | Supplement use, yes, % (n) | 209 | 43 (20.6) | 43 (20.6) | 49 (23.4) | 0.36 |
|  | Post-menopausal, yes, % (n) | 224 | 50 (22.3) | 47 (21.0) | 40 (17.9) | 0.27 |
|  | Highest education, none, % (n) | 224 | 14 (6.3) | 16 (7.1) | 12 (5.4) | 0.10 |
|  | Occupation, professional, % (n) | 224 | 35 (15.6) | 32 (14.3) | 37 (16.5) | 0.45 |
| Flavanones | Age, years | 224 | 56.3 (7.2) | 55.1 (8.0) | 53.4 (7.6) | 0.02 |
|  | BMI, kg/m^2^ | 224 | 25.4 (4.2) | 25.4 (4.3) | 25.1 (4.2) | 0.68 |
|  | Current smoker, yes, % (n) | 224 | 7 (3.1) | 10 (4.5) | 6 (2.7) | 0.55 |
|  | Physically active, yes, % (n) | 224 | 10 (4.5) | 14 (6.3) | 12 (5.4) | 0.92 |
|  | Supplement use, yes, % (n) | 209 | 49 (23.4) | 47 (22.5) | 39 (18.7) | 0.15 |
|  | Post-menopausal, yes, % (n) | 224 | 47 (21.0) | 45 (20.1) | 45 (20.1) | 0.94 |
|  | Highest education, none, % (n) | 224 | 11 (4.9) | 17 (7.6) | 14 (6.3) | 0.06 |
|  | Occupation, professional, % (n) | 224 | 34 (15.2) | 35 (15.6) | 35 (15.6) | 0.10 |
| Anthocyanins | Age, years | 224 | 55.9 (7.6) | 55.4 (8.2) | 53.5 (7.0) | 0.06 |
|  | BMI, kg/m^2^ | 224 | 25.9 (4.5) | 25.2 (4.2) | 24.7 (4.0) | 0.09 |
|  | Current smoker, yes, % (n) | 224 | 9 (4.0) | 4 (1.8) | 10 (4.5) | 0.22 |
|  | Physically inactive, yes, % (n) | 224 | 9 (4.0) | 15 (6.7) | 12 (5.4) | 0.37 |
|  | Supplement use, yes, % (n) | 209 | 46 (22.0) | 42 (20.1) | 47 (22.5) | 0.24 |
|  | Post-menopausal, yes, % (n) | 224 | 47 (21.0) | 44 (19.6) | 46 (20.5) | 0.86 |
|  | Highest education, none, % (n) | 224 | 19 (8.5) | 10 (4.5) | 13 (5.8) | 0.59 |
|  | Occupation, professional, % (n) | 224 | 33 (14.7) | 38 (17.0) | 33 (14.7) | 0.61 |
| Flavan-3-ols | Age, years | 224 | 56.2 (8.1) | 55.5 (7.8) | 53.1 (6.8) | 0.01 |
|  | BMI, kg/m^2^ | 224 | 25.5 (4.2) | 25.5 (4.3) | 24.8 (4.1) | 0.34 |
|  | Current smoker, yes, % (n) | 224 | 7 (3.1) | 9 (4.0) | 7 (3.1) | 0.83 |
|  | Physically active, yes, % (n) | 224 | 13 (5.8) | 10 (4.5) | 13 (5.8) | 0.96 |
|  | Supplement use, yes, % (n) | 209 | 44 (21.1) | 42 (20.1) | 49 (23.4) | 0.29 |
|  | Post-menopausal, yes, % (n) | 224 | 51 (22.8) | 48 (21.4) | 38 (17.0) | 0.09 |
|  | Highest education, none, % (n) | 224 | 15 (6.7) | 17 (7.6) | 10 (4.5) | 0.04 |
|  | Occupation, professional, % (n) | 224 | 37 (16.5) | 35 (15.6) | 32 (14.3) | 0.58 |
| Flavonols | Age, years | 224 | 56.7 (7.9) | 54.8 (7.8) | 53.3 (6.9) | 0.01 |
|  | BMI, kg/m^2^ | 224 | 25.6 (4.2) | 25.2 (4.4) | 25.1 (4.1) | 0.45 |
|  | Current smoker, yes, % (n) | 224 | 5 (2.2) | 9 (4.0) | 9 (4.0) | 0.45 |
|  | Physically active, yes, % (n) | 224 | 14 (6.3) | 10 (4.5) | 12 (5.4) | 0.92 |
|  | Supplement use, yes, % (n) | 209 | 47 (22.5) | 39 (18.7) | 49 (23.4) | 0.09 |
|  | Post-menopausal, yes, % (n) | 224 | 53 (23.7) | 45 (20.1) | 39 (17.4) | 0.08 |
|  | Highest education, none, % (n) | 224 | 15 (6.7) | 15 (6.7) | 12 (5.4) | 0.53 |
|  | Occupation, professional, % (n) | 224 | 36 (16.1) | 41 (18.3) | 27 (12.1) | 0.39 |
| Flavones | Age, years | 224 | 56.4 (7.9) | 55.2 (7.4) | 53.2 (7.4) | 0.01 |
|  | BMI, kg/m^2^ | 224 | 25.7 (4.1) | 25.3 (4.2) | 24.8 (4.3) | 0.23 |
|  | Current smoker, yes, % (n) | 224 | 9 (4.0) | 5 (2.2) | 9 (4.0) | 0.45 |
|  | Physically active, yes, % (n) | 224 | 9 (4.0) | 13 (5.8) | 14 (6.3) | 0.83 |
|  | Supplement use, yes, % (n) | 209 | 43 (20.6) | 49 (23.4) | 43 (20.6) | 0.88 |
|  | Post-menopausal, yes, % (n) | 224 | 44 (19.6) | 47 (21.0) | 46 (20.5) | 0.86 |
|  | Highest education, none, % (n) | 224 | 17 (7.6) | 15 (6.7) | 10 (4.5) | 0.51 |
|  | Occupation, professional, % (n) | 224 | 34 (15.2) | 37 (16.5) | 33 (14.7) | 0.32 |
| Polymers | Age, years | 224 | 55.7 (8.1) | 54.4 (7.3) | 54.8 (7.6) | 0.47 |
|  | BMI, kg/m^2^ | 224 | 25.2 (4.2) | 26.0 (4.6) | 24.6 (3.7) | 0.46 |
|  | Current smoker, yes, % (n) | 224 | 6 (2.7) | 10 (4.5) | 7 (3.1) | 0.54 |
|  | Physically active, yes, % (n) | 224 | 14 (6.3) | 13 (5.8) | 9 (4.0) | 0.77 |
|  | Supplement use, yes, % (n) | 209 | 45 (21.5) | 44 (21.1) | 46 (22.0) | 0.72 |
|  | Post-menopausal, yes, % (n) | 224 | 52 (23.2) | 41 (18.3) | 44 (19.6) | 0.17 |
|  | Highest education, none, % (n) | 224 | 14 (6.3) | 16 (7.1) | 12 (5.4) | 0.21 |
|  | Occupation, professional, % (n) | 224 | 37 (16.5) | 34 (15.2) | 33 (14.7) | 0.62 |
| Proanthocyanidins | Age, years | 224 | 55.4 (7.9) | 54.5 (7.3) | 54.8 (7.8) | 0.62 |
|  | BMI, kg/m^2^ | 224 | 25.7 (4.0) | 25.6 (4.5) | 24.6 (4.1) | 0.10 |
|  | Current smoker, yes, % (n) | 224 | 7 (3.1) | 8 (3.6) | 8 (3.6) | 0.95 |
|  | Physically active, yes, % (n) | 224 | 13 (5.8) | 13 (5.8) | 10 (4.5) | 0.70 |
|  | Supplement use, yes, % (n) | 209 | 46 (22.0) | 44 (21.1) | 45 (21.5) | 0.84 |
|  | Post-menopausal, yes, % (n) | 224 | 47 (21.0) | 41 (18.3) | 49 (21.9) | 0.33 |
|  | Highest education, none, % (n) | 224 | 15 (6.7) | 15 (6.7) | 12 (5.4) | 0.07 |
|  | Occupation, professional, % (n) | 224 | 33 (14.7) | 32 (14.3) | 39 (17.4) | 0.20 |

Values are mean (SD) or % (n) where indicated.

**Table S3: 10-year change in age-related cognitive score by tertiles of 10-year change in flavonoid subclass intake.**

| **Subclass, mg/d** | **n=** | **T1** | **T2** | **T3** | **P=** |
| --- | --- | --- | --- | --- | --- |
| Total flavonoids | 224 | 0.02 (-0.22,0.26) | -0.11 (-0.33,0.12) | 0.18 (-0.05,0.41) | 0.37 |
| Flavanones | 224 | -0.12 (-0.35,0.12) | -0.13 (-0.32,0.06) | 0.33 (0.12,0.55) | 0.01 |
| Anthocyanins | 224 | -0.13 (-0.35,0.10) | -0.10 (-0.35,0.15) | 0.32 (0.07,0.56) | 0.02 |
| Favan-3-ols | 224 | 0.00 (-0.24,0.24) | -0.09 (-0.34,0.15) | 0.18 (-0.04,0.40) | 0.31 |
| Flavonols | 224 | -0.04 (-0.27,0.20) | -0.03 (-0.25,0.19) | 0.15 (-0.07,0.38) | 0.28 |
| Flavones | 224 | -0.17 (-0.38,0.04) | 0.07 (-0.13,0.28) | 0.19 (-0.09,0.46) | 0.05 |
| Polymers | 224 | 0.05 (-0.19,0.28) | 0.02 (-0.16,0.20) | 0.02 (-0.21,0.25) | 0.88 |
| Proanthocyanidins | 224 | -0.07 (-0.31,0.16) | 0.11 (-0.09,0.31) | 0.05 (-0.24,0.34) | 0.46 |

Values are adjusted means (95% CI), n=224. Means were adjusted for 10-year change in age (years), BMI (kg/m2), current smoking (yes or no), physical activity (active, moderately active, inactive), post-menopausal status (yes or no), vitamin supplement use (yes or no) and intakes of energy (kcal/d, in tertiles), alcohol (g/d, in tertiles) and fat (g/d, in tertiles), occupation (professional, intermediate, skilled non-manual, skilled manual, partly skilled or unskilled), highest education level (no qualifications; O-Level, GCSE, NVQ2/SVQ2, or Scottish Intermediate; Scottish Higher, NVQ3, city and guilds, Pitman, A Level, Scottish Advanced Higher, or Higher Vocational training; University degree, Postgraduate degree, NVQ5, or SVQ5), verbal IQ score (NART score) and presence of learning disabilities, depression or neurological conditions (yes or no). P= p-trend calculated using ANCOVA. A positive change in cognitive score translates as improvement over 10 years and a negative score is a decline.

**Figure S1: Participant flow chart**

Attended for repeat cognitive testing after 10-years; n=224

**[included in longitudinal analysis]**

Attended for cognitive testing at the same time-point as FFQ between 1999-2017; n=1126

**[included in cross-sectional analysis]**

Attended for MRI scan; n=32

**[included in MRI analysis]**

Monozygotic twin-pairs; n=243

**[included in co-twin case-control analyses if diet-discordant]**

Dizygotic twin-pairs; n=254

Individuals; n=132

Incomplete or implausible FFQ; n=999

Completed an eligible FFQ between 1999 and 2017; n=4773

Participants; n=5772
